# Acidophilic sulphate‐reducing bacteria: Diversity, ecophysiology, and applications

**DOI:** 10.1111/1758-2229.70019

**Published:** 2024-10-13

**Authors:** Luis Felipe Valdez‐Nuñez, Andreas Kappler, Diana Ayala‐Muñoz, Idelso Jamín Chávez, Muammar Mansor

**Affiliations:** ^1^ Biotechnology, Department of Biological Sciences National University of Cajamarca. Av. Atahualpa 1050 Cajamarca Peru; ^2^ Geomicrobiology, Department of Geosciences University of Tübingen Tübingen Germany; ^3^ Cluster of Excellence: EXC 2124 Controlling Microbes to Fight Infection Tübingen Germany; ^4^ Biotechnology Engineering, Department of Engineering and Applied Sciences University of Las Américas Quito Ecuador

## Abstract

Acidophilic sulphate‐reducing bacteria (aSRB) are widespread anaerobic microorganisms that perform dissimilatory sulphate reduction and have key adaptations to tolerate acidic environments (pH <5.0), such as proton impermeability and Donnan potential. This diverse prokaryotic group is of interest from physiological, ecological, and applicational viewpoints. In this review, we summarize the interactions between aSRB and other microbial guilds, such as syntrophy, and their roles in the biogeochemical cycling of sulphur, iron, carbon, and other elements. We discuss the biotechnological applications of aSRB in treating acid mine drainage (AMD, pH <3), focusing on their ability to produce biogenic sulphide and precipitate metals, particularly in the context of utilizing microbial consortia instead of pure isolates. Metal sulphide nanoparticles recovered after AMD treatment have multiple potential technological uses, including in electronics and biomedicine, contributing to a cost‐effective circular economy. The products of aSRB metabolisms, such as biominerals and isotopes, could also serve as biosignatures to understand ancient and extant microbial life in the universe. Overall, aSRB are active components of the sulphur and carbon cycles under acidic conditions, with potential natural and technological implications for the world around us.

## Graphical abstract

Acidophilic sulphate‐reducing bacteria (aSRB) perform dissimilatory sulphate reduction under low pH and high metal conditions. They play key roles in the sulphur and carbon cycles in natural acidic environments, emphasized by their interactions with other microbial groups, which create an intricate ecological network. Potential applications of aSRB and their products include treating acid mine drainage, technological uses of metal sulphides, and applications as biosignatures.

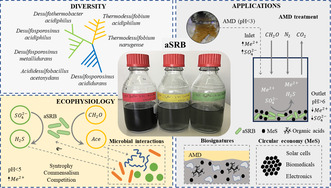

## INTRODUCTION

Acidophilic sulphate‐reducing bacteria (aSRB) belong to a very specific prokaryotic group that performs dissimilatory sulphate reduction (DSR) in acidic environments (pH < 5.0), thus contributing to sulphur cycling under these extreme conditions (Baker & Banfield, 2003; Johnson & Hallberg, 2003; Meier et al., 2004). Mining pit lakes, acid rock drainages, acid mine drainages (AMD), and acidic thermal environments are some examples of low pH habitats in which aSRB have been reported. They are usually found in anoxic zones in these environments (Alazard et al., 2010; Frolov et al., 2017, 2018; Kolmert & Johnson, 2001; Sánchez‐Andrea et al., 2013), although members of this microbial group have also been isolated or detected by molecular analyses under oxic conditions (*E*_h_ values from +74 to +450 mV), indicating their importance in widespread environmental niches (Karnachuk, Kurganskaya, et al., 2015; Valdez‐Nuñez et al., 2022). To survive in acidic environments, aSRB (and other acidophiles) are known for their resistance against high ionic strength and high concentrations of protons and heavy metals (Azabou et al., 2007; Martins et al., 2009).

Although aSRB are found at relatively low numbers in comparison to other microbial taxa (<15% of the total microbial community; Gavrilov et al., 2019; van der Graaf et al., 2020), or even as a rare biosphere in acidic environments (Hausmann et al., 2019), they play a crucial role in sulphur and carbon cycling. Acidophilic SRB couple the respiration of sulphate with organic matter degradation, specifically by using low molecular weight organic compounds (e.g., lactate, propionate, acetate, glucose, etc.) that are supplied from fermentative metabolisms (Koschorreck, 2008). Besides simple organic matter, evidence for the degradation of complex organic polymers by aSRB is increasing. The degradation of complex polymers is of vital ecological importance as it fills an important metabolic niche in these extreme ecosystems (Dyksma & Pester, 2023).

The coupling of carbon and sulphur metabolisms by aSRB generates multiple by‐products such as sulphide, acetate, and carbon dioxide, that can be used by other microbial guilds, thus driving other biogeochemical cycles (Kimura et al., 2006; Meier et al., 2004; Sánchez‐Andrea et al., 2022; van den Ende et al., 1997). The metabolic flexibility of aSRB allows for interactions (mainly syntrophic) between this group and many other microbial partners (Hausmann et al., 2016), building a network in which aSRB become key community members in acidic habitats.

Interest in applying the metabolisms of aSRB in biotechnology is especially high nowadays. Acidophilic SRB contribute to natural attenuation or intrinsic bioremediation of their own environments by decreasing the concentration of several metal/non‐metal species, through their immobilization by sulphide production (biomineralization), or by increasing the pH of their microenvironment through bicarbonate production (alkalinization; Gupta et al., 2018; Sánchez‐Andrea et al., 2012). Their metabolisms are potentially useful for wastewater remediation, resource recovery of precious metals, and production of metal sulphide nanoparticles that can be applied for technological purposes (Ayangbenro et al., 2018; Johnson & Sánchez‐Andrea, 2019; Priyadarshanee & Das, 2021). Moreover, understanding the mineral formation and organic preservation of aSRB are of interest as potential biosignatures for microbial life in acidic environments, such as those found throughout Earth and on early Mars (Amils & Fernández‐Remolar, 2020; Dopson & Johnson, 2012; Hedrich & Schippers, 2020).

Several reviews on sulphate reducers have been published in the last decades. However, those were focused mainly on either neutrophilic SRB (nSRB; Muyzer & Stams, 2008; Rabus et al., 2015) or they described aSRB in specific biotechnological applications (Ayangbenro et al., 2018; Sánchez‐Andrea et al., 2014). In this review, we aim to provide a more holistic description of the diversity and physiology of aSRB and move forward with additional overviews on their biotechnological applications and biosignature potentials. More detailed descriptions of their physiology and participation in biogeochemical cycles, including their networks with other microbial guilds, will open new research topics in microbial ecology and biotechnological applications of this prokaryotic group.

## DIVERSITY OF aSRB

Metagenomic analyses have reported the presence and even dominance of aSRB in acidic and sulphate‐rich environments. As an example, the acidic pit lake Filón Centro in the Iberian Pyrite Belt (IPB), Spain, was shown to be dominated by *Desulfomonile* sp. in the anoxic deep layer (pH from 2.9 to 4.8 and sulphate concentration of 125 mM). High concentrations of *Desulfomonile* were also reported in the IPB pit lake Cueva de la Mora at the chemocline (pH 3.9, 41 mM sulphate). Further down, in the anoxic deep layer of the same pit lake (pH 4.5, 126 mM sulphate), putative novel aSRB affiliated with *Actinobacteria*, *Chloroflexi*, and *Nitrospirae* were found in high concentrations (Ayala‐Muñoz, Burgos, et al., 2022; Ayala‐Muñoz, Macalady, et al., 2022; van der Graaf et al., 2020). Other examples include aSRB detected in sediments from the Río Tinto (Sánchez‐Andrea et al., 2012), metal‐rich streams in a sulphide mine in Huelva, Spain (Rowe et al., 2007), AMD from Carnoulès, France (Giloteaux et al., 2013), and mine tailings from a copper mine in Chile (Diaby et al., 2007).

During microbial enrichment processes and bioreactor experiments, specific aSRB genera can become abundant. Acidic microcosms (pH 3.2–3.3) using sediments collected from the acidic pit lake 111 from Brandenburg, Germany, showed an abundance of *Thermodesulfobium‐* and *Desulfosporosinus*‐affiliated 16S rRNA genes (Meier et al., 2012). The pore water of the sediments had sulphate concentrations between 9.0 and 16.2 mM and pH between 2.6 and 3.0 (Meier et al., 2012). Similarly, microcosms (pH 3.4–4.8) with sediments from tunnels polluted by AMD in Cajamarca, Peru showed the abundance of *Desulfosporosinus* and *Desulfovibrio* spp. (Valdez‐Nuñez et al., 2022). Water samples taken in these tunnels reflected a pH ranging from 2.3 to 5.4, but pore water from the collected sediments had a pH around 6.0 (Valdez‐Nuñez et al., 2022). Other enrichment processes from Arctic mine sediments with pH ranging from 3.0 to 7.0 showed the dominance of *Desulfosporosinus*, *Desulfotomaculum*, and *Desulfurospora‐*affiliated 16S rRNA genes (Dev et al., 2021). In bioreactors (pH 2.5–3.5) filled with acidic sediments (pH 2.0) of the Azufre River from Chile to treat AMD, *Desulfosporosinus* was also found in abundance (>55% of the total community; González et al., 2019). Similarly, the same *Desulfosporosinus* genus that naturally occurred at low numbers in AMD samples (0.0025%–0.0093%) from abandoned metal mine sites in Japan, became predominant (27.3%–87.0% of each total SRB‐like population) during the treatment of AMD (pH 3.4–3.7) using passive bioreactors (Sato et al., 2019).

Acidophilic SRB have been found in a diversity of acidic environments to date, although more remains to be discovered via molecular and cultivation‐based methods. So far, only a few aSRB species have been isolated (Table 1) and therefore physiologically described in depth. All the reported aSRB isolates are part of the *Firmicutes* phylum. Within the family *Thermodesulfobiacea*, *Thermodesulfobium narugense* grew on H_2_/CO_2_, and *T. acidiphilum* grew on H_2_/formate (Frolov et al., 2017). Within the family *Thermoanaerobacteraceae*, *Desulfothermobacter acidiphilus* grew on H_2_/formate (Frolov et al., 2018). Within the family *Peptococcaceae*, *Desulfosporosinus acididurans* grew on organic acids, alcohols, and sugars (Sánchez‐Andrea et al., 2015); *D. acidiphilus* and *D. metallidurans* grew on H_2_, organic acids and sugars (Alazard et al., 2010; Panova et al., 2021); and *Desulfobacillus acidavidus* grew on glycerol (Johnson et al., 2009). Furthermore, *Acididesulfobacillus acetoxydans* strain INE, an aSRB from a novel genus, grew on organic acids (Sánchez‐Andrea et al., 2022). As a whole, previously isolated aSRB display the capacity to degrade different simple organic molecules and H_2_, but not complex organic matter.

**TABLE 1:** Isolated aSRB, sample material and area from which they were isolated.

Isolated aSRB	Sample material	Area, country	pH range	Reference
*Desulfothermobacter acidiphilus*	Terrestrial hot spring	Kamchatka, Russia	2.9–6.5	(Frolov et al., 2018)
*Desulfosporosinus acidiphilus*	Acid mining effluent decantation pond sediment	Beaujolais, France	3.6–6.5	(Alazard et al., 2010)
*Thermodesulfobium acidiphilum*	Geothermally heated soil	Kamchatka, Russia	3.7–6.5	(Frolov et al., 2017)
*Desulfosporosinus acididurans*	River sediments	White river and Tinto River, Spain	3.8–7.0	(Sánchez‐Andrea et al., 2015)
*Acididesulfobacillus acetoxydans*	Acidic sediments from a dam	Tinto river, Spain	3.9–6.5	(Sánchez‐Andrea et al., 2022)
*Thermodesulfobium narugense*	Hot spring sediments	Narugo, Japan	4.0–6.5	(Mori et al., 2003)
*Desulfosporosinus metallidurans*	Microbial mat in a tailing dam at a gold mining site	Komsomolsk, Russia	4.0–7.0	(Panova et al., 2021)

## ECOPHYSIOLOGY OF aSRB

### Living under acidic conditions

**FIGURE 1:**
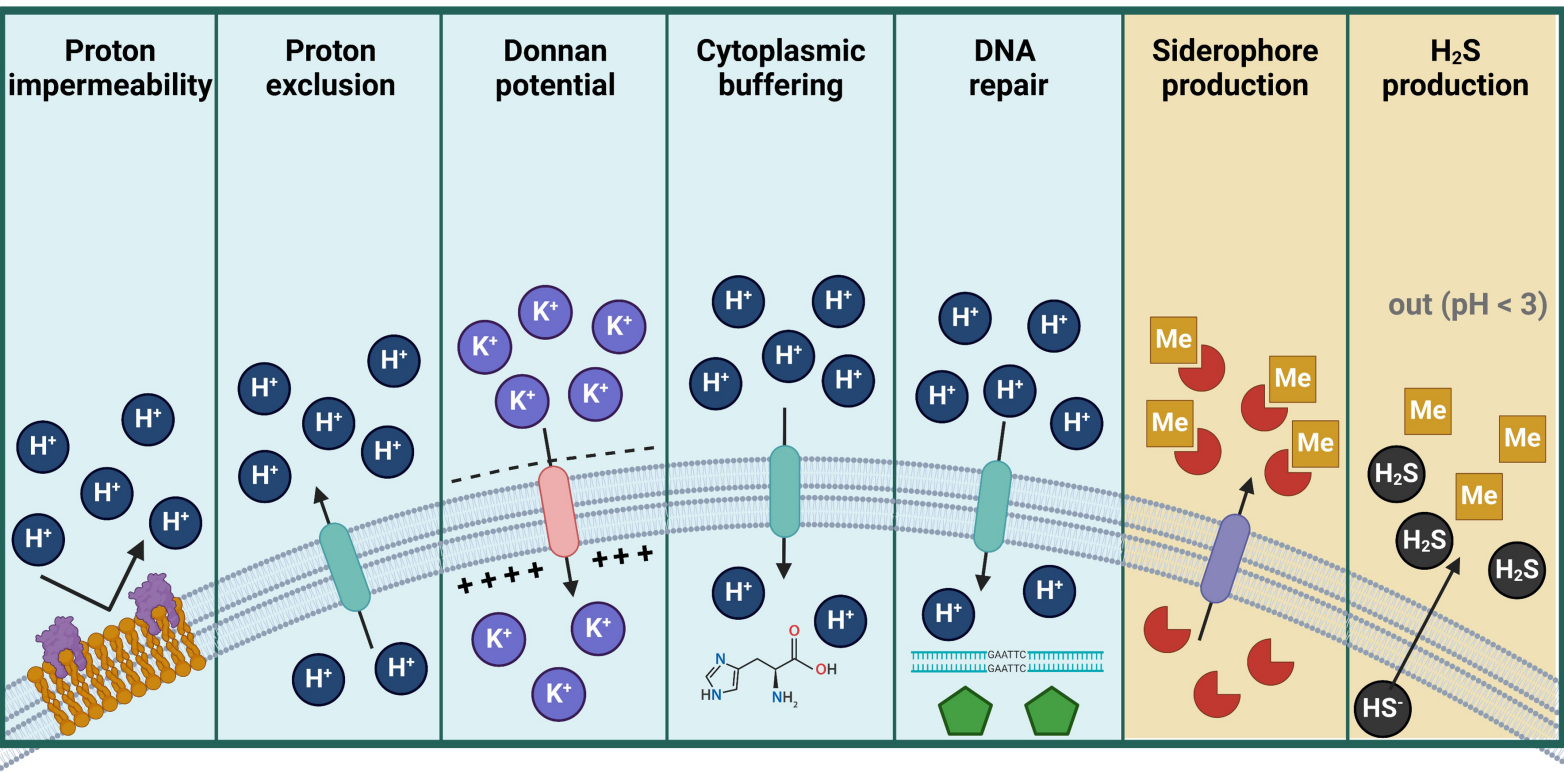
Morphological and physiological adaptations of aSRB under acidic (light‐blue background) and high metal conditions (light‐brown background). H^+^: protons; K^+^: potassium; Me: metals; H_2_S: hydrogen sulphide; yellow cell membrane: hopanoid lipids, purple‐attached proteins: Omp40/PspA proteins; structural formula: histidine; Green pentagon: chaperones; red semicircles: siderophores; +: positive charge; −: negative charge. Arrows indicates input/output of compounds throughout the cell membrane (turquoise: proton; pink: potassium; purple: siderophores) (Created with BioRender).

Given the harsh conditions that acidophiles are adapted to, unique physiological traits have been described (Figure 1). For example, acidophiles, must keep pH gradients of considerable orders of magnitude between them and their immediate environments. Acidophilic bacteria usually maintain an internal pH of around 6.0 while growing at pH lower than 3.0 (Krulwich et al., 2011). The mechanisms of pH homeostasis that acidophiles apply in general consist of proton exclusion, exchange, pumping and consumption, and cytoplasmic buffering (Zammit & Watkin, 2016). Acidophiles also have strategies for damage mitigation involving DNA repair and synthesis of acid‐stable proteins to thrive in environments with low pH and high‐metal(oid) contents (Ferrer et al., 2016). It is likely that aSRB use the same mechanisms to thrive under low pH conditions; however, more focused research (e.g., using pure cultures of aSRB) need to be addressed to support this hypothesis.

As a whole, acidophiles can decrease proton permeability by modulating components of the cell envelope. The presence of hopanoid lipids in the cytoplasmic membrane (Jones et al., 2011) or membrane proteins such as Omp40 (Guiliani & Jerez, 2000) and PspA (Kobayashi et al., 2007) are structural adaptations used for proton exclusion in acidophilic bacteria. In aSRB, similar mechanisms have been observed to maintain cell homeostasis at low pH. For instance, an increase of acyl/ether glycerol (AEG) lipids with a saturated ether moiety and branched‐chain fatty acids (e.g., iso‐C_15:0_), both related to cell resistance to low pH conditions, was found in the membrane lipid composition of *A. acetoxydans* (pH 3.9–5.0). In addition, poly‐gamma‐glutamate polymer and spermidine were also found after proteome analysis of this bacterium with potential roles in acid stress resistance (Sánchez‐Andrea et al., 2022).

Acidophiles also keep an internal positive membrane potential (Matin, 1990). Acidophiles, including aSRB, can pump cations such as K^+^ and Na^+^ into the cytoplasm to reduce the influx of protons by electrostatic repulsion (Egas et al., 2024; Jones et al., 2011; Karnachuk, Mardanov, et al., 2015; Kovaliova et al., 2017; Sánchez‐Andrea et al., 2022). Putative proton efflux systems can also directly pump protons out from the cytoplasm (e.g., H^+^/Cl^−^ exchange transporters in *A. acetoxydans*) (González et al., 2014; Sánchez‐Andrea et al., 2022), which could be useful to remove protons that originate from dissociation of low‐molecular weight organic acids upon entering the cell. Greater activity of cation pumping than proton efflux systems can generate an internal positive membrane potential using the Donnan potential mechanism (Baker‐Austin & Dopson, 2007; Sánchez‐Andrea et al., 2022).

Acidophiles could buffer cytoplasmic pH and produce low‐molecular weight chelators to avoid metal toxicity. Molecules with an abundance of alkaline amino acids such as lysine, histidine, and arginine function as buffers that help to stabilize the internal pH of acidophiles (Zammit & Watkin, 2016). Additionally, a protection mechanism against acid stress is related to the presence and increase of saturated ether‐bound lipids in the membrane, as has been reported in *A. acetoxydans* (Sánchez‐Andrea et al., 2022). Furthermore, the excretion of siderophores (functioning as metal chelators) has been proposed to aid in metal toxicity (Khan et al., 2018; Roskova et al., 2022), as reported in *Pseudomonas* species (Zawadzka et al., 2006). The presence of sulphate ions also aids via complexation of free metals (Dopson et al., 2014; Dopson & Holmes, 2014). Finally, as discovered via genomic surveys, some sulphate reducers may also have a putative siderophore export system that could aid survival under high metal concentrations (Barton et al., 2023). However, it is unclear if this is relevant at low pH where metal solubility is orders of magnitude higher than at circumneutral pH.

### Sulphate reduction at low pH

#### Sulphate uptake and metabolism

**FIGURE 2:**
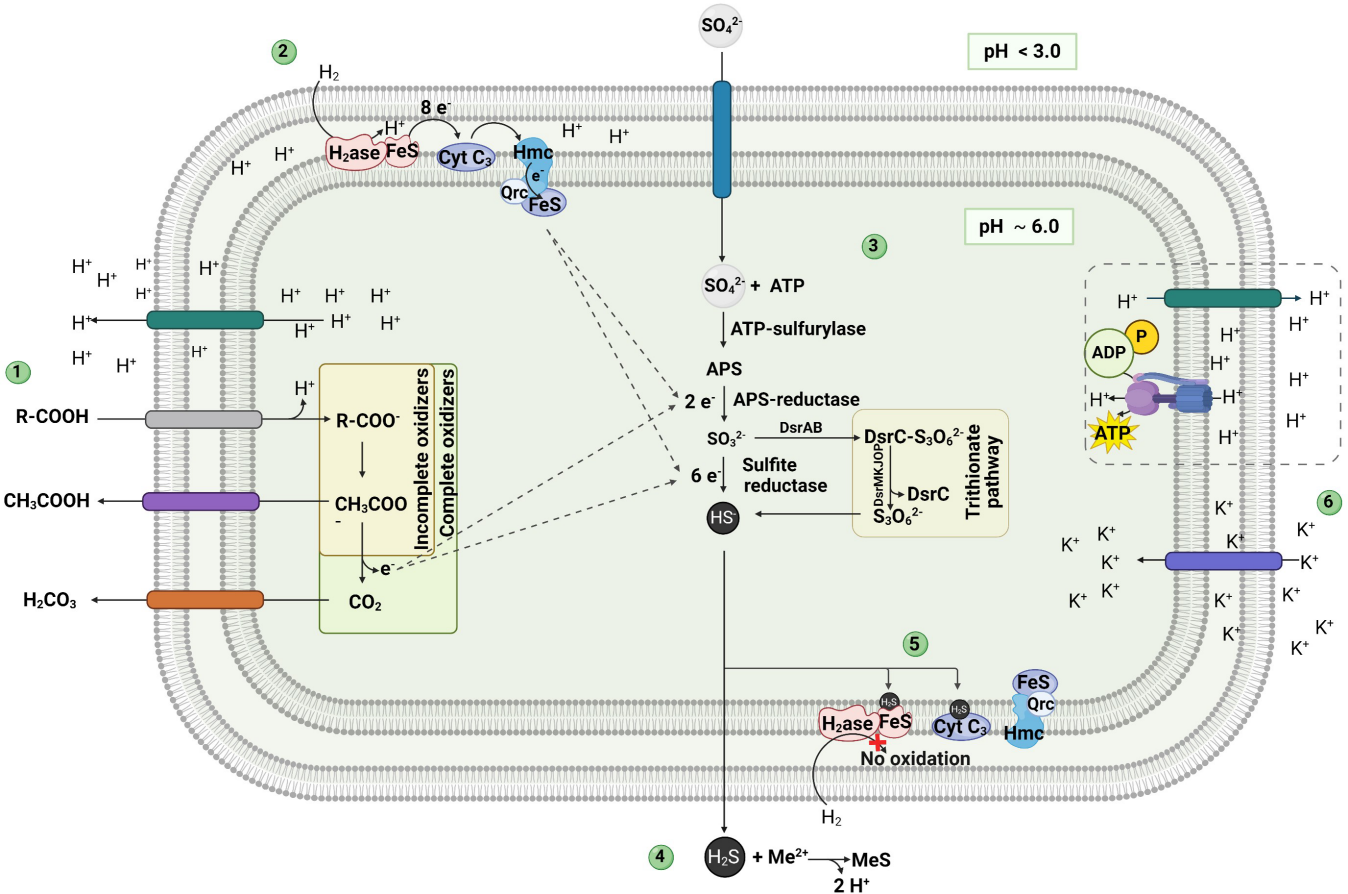
Sulphate reduction at low pH by aSRB with different electron donors and implications on sulphide and proton generation. (1) Organic electron donors occur in their protonated form outside the cell (pH <3.0). When transported intracellularly, they dissociate at the circumneutral pH of the cytoplasm (pH ~ 6.0), releasing protons. Once inside, organic electron donors are oxidized completely or incompletely to obtain electrons for sulphate reduction. (2) Inorganic electron donors such as H_2_ use *hydrogenase* and c3‐type cytochrome‐Hmc‐Qcr complexes to transfer the electrons for sulphate reduction. (3) Sulphate reduction could be performed by a common (6‐electron transfer) or by an alternative trithionate pathway (three 2‐electron transfer steps), resulting in the release of HS^−^ outside the cell. (4) H_2_S is the predominant species outside the cell due to the external pH (<3), allowing it to react with metals to produce poorly‐soluble metal sulphides (MeS). (5) Intracellular H_2_S is toxic as it can react with iron present in ferredoxin or cytochromes, inhibiting the electron transport chain. Finally, (6) protons outside the cell need to enter the cell for ATP generation by ATP synthase. However, the low proton permeability in acidophilic cells could affect ATP generation. To maintain a proton equilibrium, other ions (such as K^+^) are likely used as counter‐ions. Dashed arrows show electron transfers for sulphate reduction. Dashed square represents a hypothetical process (Created with BioRender).

Sulphate (SO_4_^2−^) is the most oxidized and most soluble form of sulphur. It is commonly used by microorganisms in either assimilatory or dissimilatory pathways (Pepper et al., 2015), with the latter being the focus in this review. DSR is performed by a highly diverse group of microorganisms under anoxic conditions, producing sulphide as a by‐product, which speciates to the toxic H_2_S gas with the typical odour of rotten eggs at pH <4 (Muyzer & Stams, 2008; Swanson et al., 2016). Because reduction of sulphate occurs in the cytoplasm (Figure 2), sulphate needs to be first transported into the cell, driven by an ion gradient (H^+^/Na^+^ antiporters) with a relatively low‐cost energy requirement (1/4 to 1/3 ATP per sulphate for sulphate transport; Rosenberg et al., 2013). Once sulphate has been taken up, it undergoes a primary activation to adenosine‐5′‐phosphosulfate (APS) by an APS sulfurylase, followed by the reduction of APS to sulphite by an APS reductase, and finally by sulphite reduction to sulphide. The specific mechanism of the last step remains under discussion because there are contrasting suggestions that this step can proceed either directly (involving a 6‐electron transfer) or gradually (involving three 2‐electron transfer steps), with the latter producing reactive intermediates such as trithionate and thiosulfate (Qian et al., 2019; Rosenberg et al., 2013). The produced sulphide is released to the outside of the cell and can react with external metals (Muyzer & Stams, 2008). Sulphide can also be re‐oxidized by some microbial guilds or abiotically via reactions with redox‐sensitive species, thus fuelling other elemental cycles (Swanson et al., 2016).

#### Electron donors

The energy available from DSR at acidic pH needs to compensate for the high energy demand of living under extreme conditions (e.g., for maintaining pH homeostasis, see Living under acidic conditions section; Baker‐Austin & Dopson, 2007; Rosenberg et al., 2013). A previous study about the influence of environmental pH on the thermodynamics of microbial redox reactions has shown that the energy gained from sulphate reduction increases with decreasing pH from a pH range of 7.0–1.0, with a secondary control on the identity of the electron donor (e.g., organics vs. H_2_; Jin & Kirk, 2018). We have extended these calculations to conditions that are more representative of an acidic, high metal, and high sulphate environment (Tables 2 and 3). The calculations show the importance of accounting for realistic environmental concentrations, speciation, and activity in determining the actual Gibbs reaction energy (Δ*G*_r_; Amend & LaRowe, 2019). This is especially true for the sulphate ion in which the activity was determined to be one fifth of the value of the concentration. In all cases, the Δ*G*_r_ values are negative (the reactions are energetically feasible) and range from −61 to −332 kJ/mol, depending on the electron donor and on whether the oxidation is complete or incomplete (Table 2).

**TABLE 2:** Gibbs energy (Δ*G*_r_) values of sulphate reduction with different electron donors under acidic (pH 3.0) and standard conditions (Δ*G*°).

Reaction	Δ*G*_r_ (kJ/mol electron donor)	Δ*G*° (kJ/mol electron donor)
1. Hydrogen	−61.40	−75.54
SO_4_^2−^ + 4H_2_ + 2H^+^ → H_2_S + 4H_2_O		
2. Propionate	−116.29	−90.53
CH_3_CH_2_COOH + 0.75SO_4_^2−^ + 1.5H^+^ → CH_3_COOH + 0.75H_2_S + H_2_CO_3_		
3. Lactate (incomplete)	−153.87	−125.88
CH_3_CHOHCOOH + 0.5SO_4_^2−^ + H^+^ → CH_3_COOH + 0.5H_2_S + H_2_CO_3_		
4. Acetate^a^	−177.98	−132.32
CH_3_COOH + SO_4_^2−^ + 2H^+^ → H_2_S + 2H_2_CO_3_		
5. Butyrate	−236.44	−184.95
CH_3_CH_2_CH_2_COOH + 1.5SO_4_^2−^ + 3H^+^ → CH_3_COOH + 1.5H_2_S + 2H_2_CO_3_		
6. Glycerol	−306.07	−280.19
CH_2_OHCHOHCH_2_OH + 0.75SO_4_^2−^ + 1.5H^+^ → CH_3_COOH + 0.75H_2_S + H_2_CO_3_ + H_2_O		
7. Lactate (complete)	−331.85	−258.20
CH_3_CHOHCOOH + 1.5SO_4_^2−^ + 3H^+^ → 1.5H_2_S + 3H_2_CO_3_		

*Note*: These values were calculated following the procedure suggested by Amend & LaRowe, 2019. Activity values (*Q*_r_) were calculated at 25°C using a modified minteq.v4 database (including lactate and glycerol) and the standard protocol of the PHREEQC 3.7.3 software (for more details, see Table 3). Free energy of formation $G_{f}^{\circ }$ values were collected from Amend & Shock, 2001.

^a^ Acetate concentration was the same (10 mM) either when it was a reactant or a product.

**TABLE 3:** Concentration and activity (*Q*_r_) values of reactants/products used to calculate Gibbs energy (Δ*G*_r_) values.

Reactants/products	Concentration (mM)	*Q*_r_
H^+^	1	1 × 10^−3^
SO_4_^2−^	50	9.98 × 10^−3^
H_2_S	1 × 10^−3^	9.72 × 10^−7^
H_2_(aq)	10	1.03 × 10^−2^
Acetate	10	9.74 × 10^−3^
Glycerol	10	1.03 × 10^−2^
Lactate	10	7.69 × 10^−3^
Propionate	10	9.82 × 10^−3^
Butyrate	10	9.80 × 10^−3^
H_2_CO_3_	0.001	1 × 10^−6^
Fe^2+^	32.5	4.17 × 10^−3^
Al^3+^	5	4.05 × 10^−5^
Mn^2+^	3	4.25 × 10^−4^
Ca^2+^	2.5	4.85 × 10^−4^
Zn^2+^	4	6.59 × 10^−4^
Cu^2+^	0.5	9.52 × 10^−5^

Different mechanisms to gain energy are employed when aSRB use inorganic (H_2_) and organic (lactate, acetate, glycerol) electron donors. On the one hand, when DSR is coupled to the oxidation of H_2_, the enzyme *hydrogenase* plays a crucial role. The generated electrons are transferred via the periplasmatic c3‐type cytochrome and Hmc and Qcr complexes and are used to reduce sulphate (Tang et al., 2021). Protons generated by the enzyme are directly involved in creating the proton motive force (pmf, an electrochemical potential produced as a result of the difference in charge between the two sides of the cell membrane [Madigan et al., 2015]), which is maintained by the extrusion of H^+^ to the outer surface of the membrane. That proton potential then drives the phosphorylation of ADP and the formation of ATP (for each SO_4_^2−^ reduced by H_2_) by the ATP synthase (Madigan et al., 2015; Qian et al., 2019; Unden, 2013; Figure 2).

On the other hand, when DSR is coupled to the oxidation of organic acids, two types of metabolisms are known: (i) complete oxidation towards CO_2_, or (ii) an incomplete oxidation with acetate being the end product (Muyzer & Stams, 2008; Rosenberg et al., 2013). When lactate is used, sulphate reducers could gain energy from substrate‐level phosphorylation (via acetyl‐CoA) producing acetate and CO_2_ (Madigan et al., 2015), and potentially also by electron‐transport phosphorylation through a pmf by using the so‐called H_2_‐cycling model. The latter involves cytoplasmatic H_2_ production, its diffusion through the cytoplasm to the periplasm, and its further oxidation as described for H_2_ as electron donor (for more details, see the H_2_‐cycling model; Rosenberg et al., 2013).

DSR performed by aSRB is an energetically favourable process. However, thermodynamics do not capture kinetics, nor the complexity associated with the enzyme machinery during the metabolism of each electron donor. The efficiency of sulphate reduction with H_2_ has been corroborated by previous studies at moderately low pH (pH4.0–4.5; Kimura et al., 2006; Meier et al., 2012; Sánchez‐Andrea et al., 2013; Valdez‐Nuñez et al., 2022). However, aSRB likely face a problem in ATP generation during electron‐transport phosphorylation with H_2_ as electron donor. This is because H_2_ oxidation generates H^+^ that subsequently need to enter the cell for ATP generation by ATP synthase, compounding the problem with proton stress at low pH. Nonetheless, acidophiles have mechanisms to deal with pH stress as previously discussed, which likely contribute to the success of their H_2_ energy metabolism.

Proton transport is the basis of respiration and energy conservation in anaerobic microorganisms because they are directly involved in creating the pmf. As explained in the previous section, acidophiles have different mechanisms to deal with the intrusion of protons, such as a low proton permeability and a reverse membrane potential of the cell membranes (Baker‐Austin & Dopson, 2007; Egas et al., 2024; Karnachuk, Mardanov, et al., 2015; Kovaliova et al., 2017; Quatrini & Johson, 2016; Sánchez‐Andrea et al., 2022). Thus, the net yield of ATP obtainable by aSRB through oxidative phosphorylation could be lower than expected. It is worth to note that Dopson et al. (2002) reported that *Acidithiobacillus caldus*, a thermoacidophile involved in the oxidation of reduced inorganic sulphur compounds, uses mainly oxidative phosphorylation to produce ATP. Their experiments suggest that intensive proton extrusion is required to maintain a proton balance inside the cell, and also that some ions (such as K^+^) can be used as a counter‐ion to obtain the same proton equilibrium in this microorganism (see Living under acidic conditions section). Similar studies with other acidophilic microorganisms were not found. Understanding how ATP is gained under low pH requires further research.

The oxidation of organic electron donors by aSRB has additional complications. Under acidic conditions, organic acids function as uncouplers of the respiratory chain because they occur in their undissociated form and can diffuse into the cell (Baker‐Austin & Dopson, 2007). Once there, the higher pH of the cytoplasm will lead to dissociation of the acid, thus releasing protons and lowering the internal pH (Figure 2; Koschorreck, 2008; Sánchez‐Andrea et al., 2014). Table 4 presents the dissociation constants (p*K*a) and speciation of organic acids and compounds relevant to acidophilic microorganisms. Despite these disadvantages, many aSRB are heterotrophs, suggesting that mechanisms for avoiding inhibition by organic acids have been developed. Furthermore, protons are also generated after organic acid oxidation, leading to the same issue of compounding proton stress as discussed for H_2_ oxidation above.

**TABLE 4:** Dissociation constants (p*K*_a_) and speciation of organic acids and compounds relevant to acidophiles at 25°C in water.

Compound	p*K*_a_	Major species below p*K*_a_	Major species above p*K*_a_
Formate	3.75	HCOOH_(aq)_	HCOO^−^
Lactate	3.86	C_3_H_6_O_3_H_(aq)_	C_3_H_6_O_3_^−^
Acetate	4.75	CH_3_COOH_(aq)_	CH_3_COO^−^
Butyrate	4.82	C_3_H_7_COOH_(aq)_	C_3_H_7_COO^−^
Propionate	4.87	C_2_H_5_COOH_(aq)_	C_2_H_5_COO^−^
Sulphide	7.02	H_2_S_(aq)_	HS^−^
Glucose	12.00	C_6_H_12_O_6(aq)_	C_6_H_11_O_6_^−^
Glycerol	14.40	C_3_H_8_O_3_	C_3_H_7_O_3_^−^

Different pathways have been proposed to circumvent the aforementioned problems related to electron transport and organic acid degradation. First, a direct pathway for electron transport has been suggested in which electrons are directly delivered from lactate oxidation to the membrane‐bound electron carrier menaquinone before being transferred to sulphate (Ramos et al., 2012; Tang et al., 2021). This direct pathway circumvents the problem with regulating H^+^ movements and might be better suited for aSRB. Secondly, a faster rate of carbon metabolism coupled to a faster rate of proton extrusion could prevent the build‐up of protons in the cytoplasm associated with organic acids metabolism (Baker‐Austin & Dopson, 2007). Different microbial species could use different mechanisms or a combination of them at different proportions.

#### Metabolic products

DSR under acidic conditions is an additionally challenging process when one considers the potential of inhibition from the build‐up of metabolic by‐products and wastes (Kaksonen & Puhakka, 2007; Koschorreck, 2008; Sánchez‐Andrea et al., 2013). After incomplete organic carbon degradation by aSRB, acetate is generated as a by‐product and is accumulated in the surrounding environment, leading to both toxicity effects and decreasing thermodynamic energy yield (Koschorreck et al., 2004). A complete degradation of acetate to CO_2_ could circumvent this problem, as has been recently reported in the isolated strain *A. acetoxydans* (Sánchez‐Andrea et al., 2022). This strain was able to grow under acetate concentrations of up to 7.5 mM (Egas et al., 2024).

Furthermore, sulphide, the final product of DSR, may also exhibit inhibitory effects on microorganisms, including aSRB. At low pH, its predominant chemical speciation (H_2_S_(aq)_) (Table 4) can pass through the cell membrane in its undissociated/acid form and may combine with iron in ferredoxin, cytochromes, and other essential iron‐containing compounds of the cell (Koschorreck, 2008). The activity of these cell components is inhibited via complexation or precipitation of the reactive centers as metal sulphides (Figure 2). High concentrations of sulphide in solution also decreases the thermodynamic energy yield available from sulphate reduction (Jin & Kirk, 2018). The negative effects of sulphide can be minimized due to its volatilization as H_2_S gas, its sequestration by metals, or by the activity of sulphide‐oxidizing microorganisms.

Bicarbonate is another by‐product coupled to the metabolism of aSRB. Bicarbonate is a proton‐consuming compound, being present predominantly as H_2_CO_3_ at low pH (Table 3; Jin & Kirk, 2018). Considering that pH is a primary control of microbial metabolisms, the alkalinization produced by bicarbonate species could change geochemical gradients and shape microbial communities in the surrounding environment (Jin & Kirk, 2018). It is worth to note that bicarbonate production by aSRB for biotechnological applications has been a matter of interest especially for biological treatments of acidic waters (Kaksonen & Puhakka, 2007; Sánchez‐Andrea et al., 2014; see Biological treatment of AMD section).

### Interaction of aSRB with metals

Living under acidic conditions, aSRB have to cope with high concentrations of metals due to their increased solubility with decreasing pH (Lewis, 2010). Many aSRB have been shown to tolerate high levels of dissolved metals, up to 236 mM of Cu^2+^, 50 mM of Fe^2+/3+^, 30 mM of Al^3+^, 8.5 mM of Ni^2+^, 8.5 mM of Co^2+^, 7 mM of Zn^2+^, and 2.7 mM of Cd^2+^ (Johnson et al., 2009; Mancini et al., 2016; Mardanov et al., 2016; Ňancucheo & Johnson, 2012; Sánchez‐Andrea et al., 2015). Resistance to metals in the *Desulfosporosinus* genus is conferred by metal‐resistance genes that code for metal‐transporting ATPases, chaperones, and efflux pumps, as well as the formation of polyphosphate granules that sequester metals prior to transportation out of the cells (Mancini et al., 2016; Mardanov et al., 2016). In addition, a metagenomic and metatranscriptomic study in the Cueva de la Mora acidic pit lake confirmed the expression of three putative metal‐resistance genes (related to Cu, Ag, and As transport and Fe storage) by the genus *Desulfomonile*, as well as the genetic potential for 16 other genes related to Al, Cu, Fe, Mn, Zn, Co, Ni, and As resistance (Ayala‐Muñoz et al., 2020). The presence of metal‐resistance genes in other aSRB remains to be elucidated.

In addition to specific intracellular mechanisms, metal toxicity is alleviated via extracellular precipitation of metals. The metabolic activities of aSRB consume net protons and generate sulphide, which lead to either the precipitation of Al hydroxides or hydroxysulphates (Falagán et al., 2017; Meier et al., 2012; Rüffel et al., 2018) or metal sulphides (MeS; for chalcophilic metals such as Fe, Zn, Ni, Co) (Reaction 1).

MeS precipitation:

(1) $$H_{2}S+{\mathrm{Me}}^{2+}➔\mathrm{MeS}+{\text{2H}}^{+}.$$

There are high interests in taking advantage of the metabolisms of aSRB for bioremediation of AMD or acidic wastewaters (for more details, see Biological treatment of AMD section). This is because dissolved sulphate and metals are removed from solution concurrent with an increase in pH. Several instances of selective metal removal in bioreactors have been summarized in Johnson and Sánchez‐Andrea (2019). Controlling the pH of the bioreactor is important in all cases as the respective mineral solubilities (*K*_sp_) are highly influenced by pH (Table 5). Minerals with lower *K*_sp_ values will precipitate at lower pH. Selective removal of Cu and Cd is achieved at pH ≤3.2, Zn, Ni, and Co at pH 4–5, and Al, Fe, and Mn at pH ≥4.5 (Bijmans et al., 2009; Falagán et al., 2017; Hedrich & Johnson, 2014; Ňancucheo & Johnson, 2012; Sahinkaya et al., 2009; Santos & Johnson, 2018, 2021; Tabak et al., 2003; Yildiz et al., 2019). The chalcophilic metals are precipitated as MeS, with Cu forming covellite (CuS) (Nancucheo et al., 2023; Santos & Johnson, 2018; Yildiz et al., 2019), Zn forming sphalerite (ZnS; Murray et al., 2017) and Ni forming a mixture of millerite (NiS), polydymite (Ni_3_S_4_), and vaesite (NiS_2_) (Bijmans et al., 2009; Yildiz et al., 2019). The mineralogy of the other precipitated MeS has not been reported, although mackinawite (FeS) or its poorly crystalline precursors (e.g., FeS_nano_) is typically the first Fe‐sulphide phase to precipitate from solution (Matamoros‐Veloza et al., 2018; Rickard & Luther, 2007) In other studies, Al precipitates as hydrobasaluminite [Al_4_SO_4_(OH)_10_·12‐36H_2_O] or felsöbányaite [Al_4_SO_4_(OH)10·4H_2_O] as determined by x‐ray diffraction (Falagán et al., 2017). Manganese has been postulated to precipitate as birnessite (MnO_2_) without confirmation via mineralogical analysis (Santos & Johnson, 2021).

**TABLE 5:** Solubility product constants (*K*_sp_) of various metal sulphides.

Mineral	Log *K*_sp_
CuS	−15.8
CdS	−8.9
ZnS	−4.5
NiS	−2.7
CoS	1.2
FeS	3.5
MnS	6.6

*Note*: Assuming simplified reactions of MeS + 2H^+^ ➔ Me^2+^ + H_2_S_(aq)_.

*Source*: From Mansor et al. (2020) and Wilkin and Beak (2017).

In some cases, selective metal removal may be complicated due to the natural complexity of the water that contains many dissolved metals at similar concentrations. For example, Ni unexpectedly precipitated together with Cu and Cd at pH 3.2 in the first bioreactor stage as an undefined MeS mixture (Hedrich & Johnson, 2014). In these cases, it may be more realistic to consider the precipitation of mixed‐metal sulphides. Examples include: nano‐chalcopyrite (CuFeS_2_) in riverbed sediments influenced by mining residues (Hochella et al., 2005), Cu‐containing mackinawite (FeS) in mine tailings (Fortin & Beveridge, 1997) and Cd‐containing wurtzite (hexagonal ZnS) and arsenic‐containing Cu sulphides in anoxic water columns of acidic pit lakes (Sánchez‐España et al., 2020; van der Graaf et al., 2020). In all cases, the H_2_S production is attributed to SRB living across a wide pH range (acidic to increasingly near‐neutral with depth). Mixed‐metal sulphides are also known to form in cultures of neutrophilic SRB (nSRB), either in the form of solid solutions or nano‐sized inclusions (Mansor et al., 2020; Mansor, Berti, et al., 2019; Mansor, Winkler, et al., 2019). Similar investigations for aSRB are lacking. Association of metals via adsorption to minerals also cannot be ruled out, as most metal sulphides have low points of zero charge (Bebie et al., 1998; Kosmulski, 2020) that allow binding of metal ions to the negatively charged mineral surfaces even at low pH. Determining the exact mineralogy, particle size, surface area, surface charge and association of the precipitated metals down to the nanoscale level will be important in determining their reactivity and stability for long‐term bioremediation as well as for designing strategies for resource recovery of precious metals.

### Interactions of aSRB with other microbial groups

Microbial interactions (e.g., syntrophy and competition) are the basis of success for prokaryotic life in many environments (Johnson, 1998; Pinheiro et al., 2023). ‘All for one and one for all’, an iconic quote of Alexandre Dumas, should perfectly represent how microbes support each other by expanding their metabolic potential in a community (Swanson et al., 2016). Typical microbial interactions involve syntrophy (population 1: +; population 2: +), commensalism (population 1: +/−; population 2: +) and competition (population 1: −; population 2: −). Although each interaction could occur between two or more microbial populations, the whole interactive network spans across different microbial communities, allowing for mutual survival.

#### Syntrophy

**FIGURE 3:**
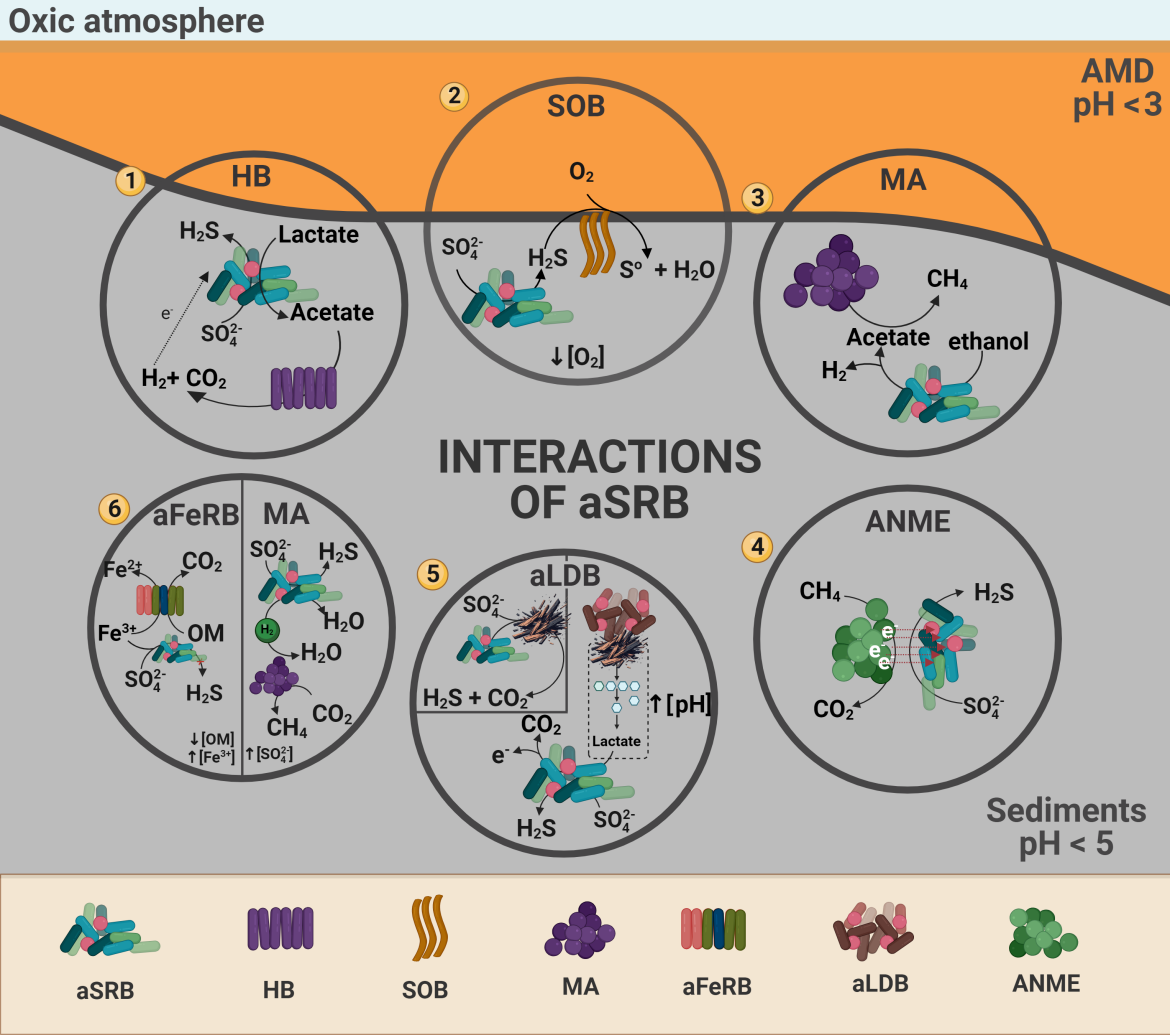
Interactions of acidophilic sulphate‐reducing bacteria (aSRB) with different microbial groups. (1) Heterotrophic bacteria (HB) metabolize acetate that is released after lactate/glycerol oxidation of aSRB, thus decreasing its concentration. In return, the former bacteria produce H_2_, which is a suitable electron donor for aSRB. (2) Sulphur‐oxidizing bacteria (SOB) oxidize H_2_S with O_2_, thus generating sulphate and low O_2_ conditions that are suitable for the development and activity of aSRB. (3) aSRB could develop a collaborative behaviour with methanogenic archaea (MA). The former bacteria produce acetate after incomplete ethanol oxidation, which is a suitable carbon source for MA. (4) Anaerobic methanotrophic archaea (ANME) transfer electrons from methane oxidation to aSRB for sulphate reduction. (5) Biodegradation of complex organic matter could be performed in a direct and indirect way; the former requires that aSRB have the metabolic pathway for polymeric material degradation and the latter involves a multi‐step process carried out by other microbial guilds such as acidophilic lignocellulose‐degrading bacteria (aLDB). (6) Acidophilic Fe(III)‐reducing bacteria (aFeRB) and aSRB could compete for specific carbon sources and Fe^3+^ as terminal electron acceptor. MA and aSRB can compete for electron donors (e.g. H_2_) under high sulphate conditions. Interactions 1, 3, 5, and 6 were corroborated by culture experiments and interactions 2 and 4 by molecular‐based data (Created with BioRender).

Syntrophy is defined as an optional and mutually beneficial interaction between two different types of microorganisms, for example between aSRB and heterotrophic bacteria. In the laboratory, Kimura et al. (2006) found that *D. acididurans* and *Acidocella aromatica* were enriched together in bioreactors (pH 4.0) and that both microorganisms benefited from this association. *D. acididurans*, an aSRB that normally oxidizes substrates to acetate as a by‐product, grows with *A. aromatica*, a heterotrophic bacterium that metabolizes acetate, thus maintaining a low concentration of this organic acid, stopping eventual toxicity for its partner. Furthermore, *Acidocella*'s metabolism is linked to H_2_ production, a suitable electron donor for *D. acididurans* (Jones et al., 2013; Koschorreck, 2008; Figure 3). The same association has been observed in microcosms and bioreactors inoculated with acidic sediments from an abandoned mining tunnel in Peru (pH 5.8) and the Azufre River in Chile (pH 2.0), respectively (González et al., 2019; Valdez‐Nuñez et al., 2022).

Sulphur‐oxidizing bacteria (SOB) and aSRB also likely interact positively with one another (Figure 3). Sulphur oxidation can proceed photoautotrophically or chemoautotrophically, with the last being more predominant in acidic environments (Pepper et al., 2015). SOB (e.g., *Thiovirga* spp.) can oxidize sulphur compounds that are by‐products of sulphate reducers (e.g., H_2_S), thus regenerating sulphate and contributing to sulphur recycling under acidic conditions (Ly et al., 2019; Meier et al., 2004; Swanson et al., 2016; van den Ende et al., 1997). In addition, when sulphur oxidation is coupled to oxygen reduction, oxygen is consumed, thus generating low O_2_ conditions suitable for the development and activity of aSRB (van den Ende et al., 1997). A similar scenario has been observed in the water column of acidic mine pit lakes in the IPB, in which anaerobic microbial communities (including aSRB) develop after oxygen consumption promoted by aerobic microorganisms (Puente‐Sánchez et al., 2014; van der Graaf et al., 2020). It is worth mentioning that the SOB need to position themselves at the optimal interface for H_2_S and O_2_, as observed with *Beggiatoa* and some bacteria of the family *Desulfobulbaceae* (Swanson et al., 2016). This relationship would likely also be relevant in acidophilic microbial mats, in which an active cycling of sulphur species is reported (Prieto‐Barajas et al., 2018). Interactions between microbial guilds of the sulphur cycle—demonstrating a strong relation between reductive and oxidative metabolisms—could be defined as syntrophy (van den Ende et al., 1997).

At moderately low pH conditions (pH 4.0–5.0), a collaboration between sulphate reducers and methanogenic archaea has been reported. In an anaerobic batch reactor developed for the treatment of a synthetic AMD, it was found that *Syntrophobacter*, a sulphate reducer with an incomplete ethanol oxidizing metabolism, produced acetate and promoted the establishment of *Methanosaeta*, an acetoclastic methanogenic archaeon that was able to metabolize acetate to methane and carbon dioxide. This created a syntrophic, mutually beneficial interaction between them (Giordani et al., 2019). A previous research reported the same archaeal genus in sediments of Río Tinto adjacent to where aSRB have been isolated (Sanz et al., 2011). It is worth to mention that the maintenance of this cooperation could be possible even if ethanol is abse. The same synergistic‐like interaction between these two microbial groups has also been reported under sulphate‐depleted conditions. In this case, the ‘sulphate reducer’ switched to a fermentative lifestyle rather than continuing with sulphate reduction as their main energy metabolism (Plugge et al., 2011). Although the latest interaction has been reported at neutral pH condition, its occurrence at lower pH could be possible but it has not been confirmed.

The collaboration between sulphate reducers and anaerobic methanotrophic archaea (ANME) is another example of a well‐established positive microbial interaction (Swanson et al., 2016). Both microbial guilds are involved in interspecies extracellular electron transfer (EET) as a strategy to live in syntrophy, in which electrons from methane oxidation performed by ANME are passed on for sulphate reduction (Caldwell et al., 2008; Cui et al., 2015; Qian et al., 2019; Scheller et al., 2016). The occurrence of this collaboration in acidic environments has only been hypothesized based on molecular‐based assays (Ni et al., 2018; Yanagawa et al., 2013; Figure 3). The EET mechanism has the potential to link electrons and energy between acidophiles.

It is interesting to speculate on how electron transfer between microbial guilds may proceed differently at acidic pH than at circumneutral pH. Electron transfer is known to proceed via several mechanisms including direct contact, microbial nanowires, organic‐based electron shuttles, and through redox active moieties in extracellular polymeric substances and (conductive) minerals (Kappler et al., 2021; Mansor & Xu, 2020). The functional groups associated with electron transfer via organic components must be different at acidic pH than at circumneutral pH, as dictated by the stability of organic molecules at different pH values. Similarly, the mineral assemblages involved in electron transfer should be different at different pH values as dictated by mineral solubilities. Redox‐active and mixed‐valent Fe minerals such as nanoparticulate magnetite for example have been shown to play a crucial role in mediating electron transfer between microbial guilds at circumneutral pH (summarized in Mansor & Xu, 2020). The importance of nano‐magnetite at acidic pH is; however, questionable given that magnetite is more soluble at this pH. Other minerals, for example, the redox‐active and less soluble Cu‐containing sulphide minerals (Table 5), may play a more important role under acidic conditions.

#### Commensalism

Commensalism is defined as an interaction in which one population benefits while the other neither benefits nor harm. An example is the interaction between fermenters and aSRB. Microcosm experiments suggested that fermenters are necessary to first colonize and create conditions suitable for the subsequent activity of aSRB. This was hypothesized due to a pH increase in the microcosms before sulphide production, signifying bicarbonate ion generation by the fermenters (Valdez‐Nuñez et al., 2022). Thus, commensalism may be a survival method for aSRB under some conditions.

**FIGURE 4:**
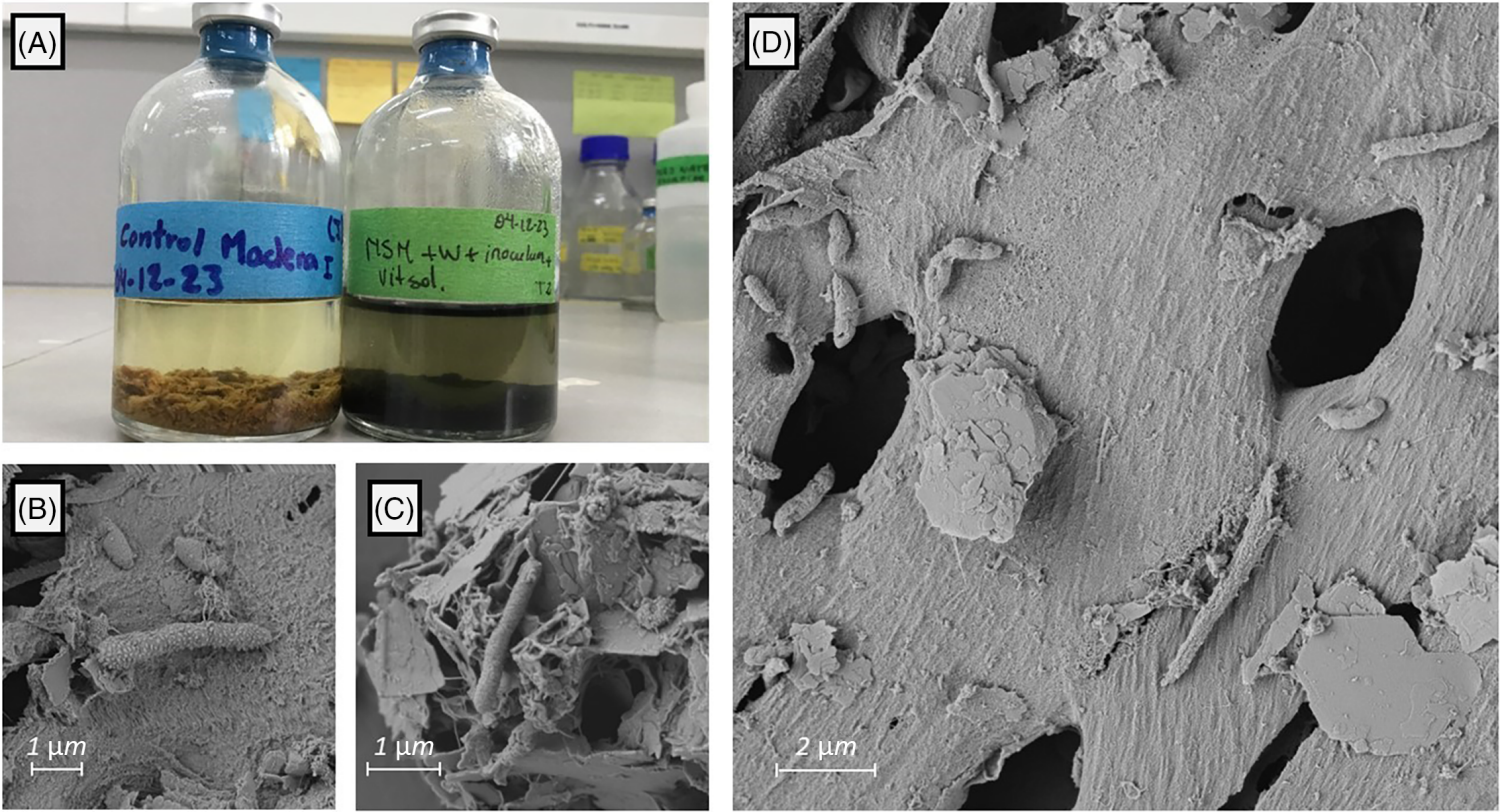
Microcosm experiment using acidophilic sulphate‐reducing bacteria (aSRB) and wood chips as the sole carbon source. (A) On the left, an abiotic control containing mineral salts medium (MSM) and wood chips collected from a Peruvian mining tunnel (Hualgayoc‐Cajamarca) with acid mine drainage (pH ~1.5). On the right, test bottle with MSM, the same wood chips and an aSRB consortium. Notice the blackish colours in this bottle indicating metal sulphides (MeS) precipitation, a product of sulphate reduction. This experiment started at pH ~3.7 and reached a pH of 5.6 after 15 days of incubation. (B–D) Scanning electron microscopy (SEM) images of wood chips from the microcosm experiment after 45 days of incubation. Notice the wood structure (lignocellulosic material) and the bacterial colonization (encrusted cells in B) on the wood surface. SEM samples were prepared with a focus on preservation of cellular structure using glutaraldehyde fixation, as detailed in Bronner et al. (2023).

Ongoing experiments also suggest that the presence of particular substrates play crucial roles in the development of aSRB populations. In particular, natural wood chips found in microcosm experiments have been observed as a good substrate for the growth of aSRB at low pH, detectable by the development of black colour (indicative of MeS precipitation) on the surface of the wood chips (Figure 4). To explain this, two ways for degradation of wood chips, which are mainly composed of organic polymers such as lignin and cellulose, could be hypothesized: direct and indirect. Direct degradation requires that aSRB have the metabolic pathway for lignocellulose degradation. Very recently, the metabolic capability for pectin (a polymer similar to lignin) biodegradation has been reported in acidophilic *Acidobacteria* with DSR capability, suggesting the existence of putative pathways for the degradation of polymeric materials under acidic pH (Dyksma & Pester, 2023). Alternatively, an indirect pathway requires a multi‐step process carried out by other microbial guilds such as acidophilic lignocellulose‐degrading bacteria (aLDB; Muyzer & Stams, 2008). The pathway therefore may take the following sequence: (i) hydrolysis of lignocellulosic material (wood chips) by aLDB, (ii) fermentation of by‐products from lignocellulose degradation, (iii) alkali generation by alternative microbial metabolisms other than sulphate reduction, and finally (iv) sulphate reduction by aSRB. A key distinction here is that this viewpoint suggests that aSRB cannot tolerate truly acidic pH, which is of importance for bioremediation. Therefore, a microbial consortium is more tolerant over a wide pH range, which might be important to consider for effective bioremediation of acidic waters.

#### Competition

Competition is a negative interaction in which both microbial populations attempt to metabolize the same substrate(s) to survive. Under sulphate‐rich conditions, sulphate reducers normally out‐compete other microbial guilds such as methanogenic archaea for the utilization of organic carbon (Plugge et al., 2011). Acidic conditions could influence this competition. The few studies in which methanogenic archaea have been reported in acidic environments and the optimal growth pH of isolated strains (e.g., *Methanoregula boonei*, optimum pH of 5.0; Bräuer et al., 2011) suggest that cell stress caused by low pH seriously affect these microorganisms, even though the available energy of methanogenesis remains largely unchanged under acidic conditions (thermodynamic calculations at pH 4.0 considering acetoclastic methanogenesis; Jin & Kirk, 2018; Sanz et al., 2011; van der Graaf et al., 2020). In contrast, factors such as the high concentration of heavy metals in the acidic environment might shape another scenario, changing the competition to benefit methanogenesis instead aSRB (Puente‐Sánchez et al., 2014). Such competitions are important when considering the fate of organic carbon for greenhouse gas emission (e.g., released as CO_2_ or CH_4_) and the fate of nutrients (e.g., limiting heterotrophic Fe(III) reduction, which then limits the release of nutrients associated with the minerals).

aSRB could compete with other microbial partners such as acidophilic Fe(III)‐reducing bacteria (aFeRB; Figure 4). Heterotrophic and/or chemolithoautotrophic bacteria (*Acidiphilium* spp. and *Acidithiobacillus* spp.) and archaea (*Ferroplasma* spp.) are commonly found in acidic sediments (Chen et al., 2016; Meier et al., 2004). When Fe^3+^ is available in the environment, aFeRB could easily outcompete aSRB (Koschorreck, 2008), especially if specific carbon sources are present (e.g., ethanol that is used by *Acidiphilium cryptum*; Meier et al., 2004). Alternatively, it was hypothesized that aSRB and aFeRB do not need to compete with one another as the energy in the environment is always greater than the sum of the energy for thermodynamic maintenance of both microorganisms (Ling et al., 2015). It is worthwhile to note that some aSRB members (e.g., *Desulfosporosinus*) can use Fe^3+^ as an alternative electron acceptor, thus further increasing the likelihood of competition between aSRB and aFeRB (Rosenberg et al., 2013; Sánchez‐Andrea et al., 2015). Therefore, the most likely interaction between aFeRB and aSRB is via competition, although it is unclear to what extent this occurs.

## BIOTECHNOLOGICAL APPLICATIONS AND BIOSIGNATURE POTENTIALS OF aSRB

### Biological treatment of AMD

AMD is a by‐product of mining activities and is characterized by its high level of metals, sulphate, and acidity (pH <3; Dold 2014; Skousen et al., 2018). AMD generation involves chemical reactions that occur first with the oxidation of metal sulphides (e.g., pyrite (FeS_2_), arsenopyrite (FeAsS), or pyrrhotite (Fe_(1−x)_S))—which are exposed to oxygen and water during ore extraction—and with subsequent ferrous iron and sulphate production (Reaction 2). Subsequently, ferrous iron can be oxidized by oxygen (Reaction 3) and the ferric iron produced can oxidize pyrite again at a much faster rate (Reaction 4). As pH decreases (pH < 3), acidophilic iron‐oxidizing microorganisms catalyse ferrous iron oxidation and accelerate it dramatically, thus releasing more iron, sulphur, and additional metals (e.g., Zn, As, Cd, Cu) and protons into the solution (Baker & Banfield, 2003; Baker‐Austin & Dopson, 2007; Hedrich & Schippers, 2020; Rimstidt & Vaughan, 2003; Schippers, 2004; Singer & Stumm, 1970). AMD can cause toxicity in soils and waters when it is discharged into the environment (Larsson et al., 2018; Macklin et al., 2023). Because of the chemical nature of its components, the volume generated, and the high cost of neutralizing agents, AMD treatment is currently one of the biggest challenges in the mining industry.

(2) $${\mathrm{FeS}}_{2}+3.{\text{5O}}_{2}+H_{2}O\to {\mathrm{Fe}}^{2+}+{{\text{2SO}}_{4}}^{2-}+{\text{2H}}^{+},$$

(3) $${\mathrm{Fe}}^{2+}+0.25O_{2}+H^{+}\to {\mathrm{Fe}}^{3+}+0.{\text{5H}}_{2}O,$$

(4) $${\mathrm{FeS}}_{2}+14{\mathrm{Fe}}^{3+}+{\text{8H}}_{2}O\to 15{\mathrm{Fe}}^{2+}+{{\text{2SO}}_{4}}^{2-}+16H^{+}.$$

Biological treatment, either via bioaugmentation (the addition of specific microorganisms) or biostimulation (the addition of nutrients; Pepper et al., 2015), offers a promising alternative to treat AMD compared with other methods (Ayangbenro et al., 2018; Rambabu et al., 2020). Acidophilic SRB could be a key biological player for AMD bioremediation (Hedrich et al., 2018; Ňancucheo & Johnson, 2012; Santos & Johnson, 2022). These microorganisms use sulphate as an electron acceptor, thus offering the elimination of high levels of sulphate in the solution (concentrations reported up to 400 mM; Sánchez‐España et al., 2020). Furthermore, the activity of SRB produces alkalinity that neutralizes the acidity in AMD through the production of bicarbonate ions (Reaction 5) (Ayangbenro et al., 2018; Kaksonen et al., 2004). Sulphate reduction is also a proton‐consuming reaction when hydrogen is involved (Reaction 6) (Muyzer & Stams, 2008; Rabus et al., 2015). The biogenic sulphide released from sulphate reduction can react with various heavy metals (Zn^+2^, Cu^+2^, Ni^+2^, Co^+2^, Fe^+2^, or Pb^+2^) in the aqueous phase, promoting metal precipitation as metals sulphides due to their low solubility (Hedrich & Johnson, 2014; Ňancucheo & Johnson, 2012; see Interaction of aSRB with metals section, Kaksonen & Puhakka, 2007).

(5) $$\begin{array}{l} {\mathrm{CH}}_{2}{\text{OHCHOHCH}}_{2}\mathrm{OH}+0.{{\text{5SO}}_{4}}^{2-}\to {\mathrm{CH}}_{3}\text{COOH}+0.\\ {\text{5H}}_{2}S+H_{2}{\mathrm{CO}}_{3}+H^{+}, \end{array}$$

(6) $${\text{4H}}_{2}+{{\mathrm{SO}}_{4}}^{-2}+{\text{2H}}^{+}\to H_{2}S+{\text{4H}}_{2}O.$$

Depending on the chemical features of the AMD of interest, aSRB have been used/stimulated in different ways for treatment. For example, passive treatments (such as permeable reactive barriers or wetlands) that involve enhancement of the microbial activity in aquifers through substrate injections (Kaksonen & Puhakka, 2007) have been shown to be effective in stimulating aSRB for sulphate elimination, metal precipitation, and pH increase (Ilin et al., 2022). A combination of various treatment systems has been suggested to improve treatment efficiency (Clyde et al., 2010). Furthermore, the composition of the injected substrate is crucial for the development of aSRB. The so‐called ‘reactive mixtures’ composed of organic and inorganic materials have particularly been shown to be effective by producing alkalinity (gravel, calcite, limestone, and silica sand) or by yielding directly organic substrates for sulphate reducers (farm manures, compost, and wood chips; Anungstri et al., 2023; Kijjanapanich et al., 2012; Vasquez et al., 2016, 2018).

In contrast to passive treatments, active treatments such as sulphidogenic bioreactors offer a better performance of aSRB due to better control of the physicochemical parameters (Johnson & Hallberg, 2005; Kaksonen & Puhakka, 2007; Sánchez‐Andrea et al., 2014). Two types of operational designs have been used for biosulphidogenic purposes: (i) two‐stage and (ii) one‐stage, differentiated based on the precipitation sites of the metal sulphides (in‐line vs. off‐line) and their characteristics (e.g., size) (Kaksonen & Puhakka, 2007; Sánchez‐Andrea et al., 2014). In these bioreactor operations, immobilization of aSRB in porous sterile glass beads (biofilms carriers) is preferred. Different carrier materials (granulated biomass) or no packing are also used with other reactor types (e.g., up‐flow anaerobic sludge blanket bioreactor) (Kolmert & Johnson, 2001; Sampaio et al., 2020; Sánchez‐Andrea et al., 2014; Santos & Johnson, 2018). Such bioreactors could be vastly improved using inocula of high‐quality cultures of aSRB, defined as those having a wide pH and heavy metal tolerance as well as associated with easy and reproducible growth.

The establishment of aSRB cultures for bioremediation is not without its challenges, due to their sensitivity to extremely low pH and high heavy metal concentrations. Nevertheless, some surveys have reported the successful use of aSRB for synthetic/real AMD treatment at pH values from 2.0 to 5.0 using bioreactors with typical electron donors such as ethanol (Ucar et al., 2011), glycerol (Dev et al., 2021) and complex substrates like lignocellulose (Becerra et al., 2009) and waste water from Fischer‐Tropsch process (Magowo et al., 2020). The resultant effluents had pH values between 6.0 and 7.0, sulphate removal rates between 50 and 99% and high levels (>95%) of metal precipitation (Fe, Mn, Cu, Zn, Al, etc.) at the end of the treatment (Dev et al., 2021; Frederico et al., 2022; Luptáková et al., 2016; Sampaio et al., 2020; Senko et al., 2009).

Instead of pure cultures, the use of well‐adapted microbial consortia has been recently explored as a possible way to enhance sulphate reduction in AMD treatment. A microbial consortium is defined as a two‐ or more‐membered association of bacteria, performing specific metabolic processes and usually living synergistically (Madigan et al., 2015). In theory, microbial consortia systems are more efficient for biodegradation of environmental pollutants in comparison to single strains since the former has multiple redundant functionalities and robust characteristics (e.g., metal‐oxidizing/−reducing microorganisms that enhance metal removal through division of functions; Qian et al., 2020). In an AMD bioremediation system, members of the microbial consortium can have defined and specific roles, such as organic acid oxidation, sulphate reduction, and iron(III) reduction comprising several taxa and/or different genotypes of a single taxa for those functions, thus increasing the effectiveness of pollutant elimination (Chen et al., 2016). Many potential sulphidogenic microbial consortia enriched from acidic/high metal content sediments have been used for AMD treatment (Dev et al., 2021; González et al., 2019; Gupta & Sar, 2020; Le Pape et al., 2017; Ňancucheo & Barrie Johnson, 2014; Ňancucheo & Johnson, 2012). High‐rate or even complete sulphate/metal removal is accomplished (e.g., a consortium composed by *Desulfoporosinus* and *Clostridium*, which removed >80% of sulfate, and a consortium composed by *Desulfoporosinus*, an *Actinobacterium* strain and *Acidithiobacillus*, which removed >97% of soluble cooper in a synthetic AMD; Frederico et al., 2022; Santos & Johnson, 2018), confirming that microbial consortia utilization is an improved form for AMD treatment compared with monocultures.

### Circular economy based on sulphide nanoparticles recovered from metal‐rich wastes

Recovery and further utilization of MeS from metal‐rich wastes are highly desirable to offset the costs of remediation and for the initiation of a sustainable circular economy (e.g., Johnson et al., 2020). Bioreactor operations for optimal MeS recovery and downstream applications have been reviewed recently, in which MeS applications in the field of solar cells, biomedicals, electronics, and environmental remediation of toxic organic and inorganic compounds have been highlighted (Kumar et al., 2021). Recent studies have demonstrated that acidic sulphidogenic consortium can be used to synthesize ruthenium/palladium sulphide nanoparticles that are utiltized to generate high value organic components (e.g., 2, 5‐dimethyl furan, ethyl cinnamate) useful in industry (Mikheenko et al., 2019, 2022). These nanoparticles outperformed commercial ruthenium/palladium catalyst and those synthesized by the nSRB *Desulfovibrio desulfuricans*. More recently, Nancucheo et al. (2023) reported the recovery of CuS from a real AMD wastewater and its subsequent potential for photodegradation of organic dyes and as antibacterial agents and semiconductors. An earlier study also reported the recovery of Zn as ZnS from sulphidization of a real AMD, with potential application as quantum dots (Murray et al., 2017). In both studies, the AMD‐recovered MeS have similar properties to synthetic MeS from simple systems, leading to similar reactivities and an increase in confidence that a circular economy framework can be built upon.

### Biosignatures

The detection of life in the universe remains one of the key research fields that will have a profound influence on our understanding of humankind's place in the universe. Because it is generally considered that complex life will have higher barriers towards its evolution, the search for traces of life (i.e., biosignatures) has focused on simpler life such as bacteria and archaea. Their small sizes pose challenges for direct fossil detection, but their versatility and high metabolic rates confer disproportionately large impacts on their surrounding environments (Domagal‐Goldman et al., 2016). Biosignatures specific to aSRB could be particularly relevant in acidic environments such as those proposed to be present on ancient Mars (Amils & Fernández‐Remolar, 2020) and on acid rock drainages proposed to be widespread on the early Earth directly ~2.4 billion years ago, directly after the Great Oxidation Event that accelerated terrestrial pyrite weathering (Konhauser et al., 2011).

There are many types of potential biosignatures including microbialitic structures, carbonaceous matter, biominerals, stable isotopes, and trace metals (Runge et al., 2023). Studies on modern analogues, such as acidic rock drainages, hot springs, fumaroles, solfataras, hydrothermal sites and acid sulphate soils are helping to evaluate useful biosignatures (Amils & Fernández‐Remolar, 2020). These sites harbour aSRB especially under anoxic conditions. The first indicator of aSRB's activity is the presence of black sedimentary layers corresponding to the presence of MeS (nearly all the listed MeS in Table 5 are different shades of black, with the exception of the whitish ZnS). Since an estimated 97% of sulphide produced in low temperature environments is attributed to microbial sulphate reduction (Picard et al., 2016), the formation of MeS is a strong indicator for SRB's activity. Their activity can be further deduced by the decrease of sulphate and increase of acetate at the same sedimentary depth, corresponding to sulphate reduction coupled to incomplete oxidation of organic carbon (see Sulphate Reduction At Low Ph section). Finally, the direct presence of aSRB (assuming DNA‐based life) can be confirmed by microbial community analysis such as fluorescence in situ hybridization and omics approaches (Sánchez‐Andrea et al., 2012).

Studies focused on biosignatures of aSRB are relatively scarce. Modern aSRB are not known to form obvious microbialite‐like structures. Carbonaceous matter in the form of lipid biomarkers such as phytane, branched fatty acids (e.g., i/a‐15:0, i/a‐17:0, i/a‐15:1) and monounsaturated fatty acids (e.g., 16:1ω5, 17:1, 18:1ω5) are detectable in acidic environments and have been attributed to aSRB (Bühring et al., 2012; Fang et al., 2007; Pei et al., 2019; Sánchez‐García, Carrizo, et al., 2020; Sánchez‐García, Fernández‐Martínez, et al., 2020). However, their specificities to aSRB are questionable and they may instead be a general indicator for bacteria, or at most, anaerobic bacteria (Duda et al., 2016; Kaneda, 1991). A recent study has nonetheless proposed that high levels of phosphocholine lipids with mixed acyl/ether glycerol core structures could be attributed to the aSRB *Desulfomonile* in acidic pit lakes (van der Graaf et al., 2020).

As mentioned before, aSRB also promotes the precipitation of biominerals such as MeS and Al hydroxides/hydroxysulphates. These biominerals could be useful as biosignatures, especially if they are closely associated with cell‐derived organic carbon. In acidic mine tailings, mackinawite and pyrite are formed in sulphate‐reducing zones, with close association inferred between mackinawite and the cell walls (Fortin & Beveridge, 1997). In acidic pit lakes, sulphide production by aSRB resulted in the formation of wurtzite (hexagonal ZnS), digenite (Cu_1.8_S), djurleite (Cu_1.96_S), and chalcocite (Cu_2_S), as confirmed by transmission electron microscopy coupled to scanning area electron diffraction (Sánchez‐España et al., 2020; van der Graaf et al., 2020). In laboratory cultures, aSRB can form mackinawite (Fortin & Beveridge, 1997; Meier et al., 2012; Rüffel et al., 2018) and greigite (Fe_3_S_4_; Bertel et al., 2012), although their association with the cell walls are less clear. In contrast, the precipitation of Al hydroxide globules mediated by enrichments or isolates of *Thermodesulfobium* spp. is particularly intriguing as close association with the cell surfaces is visible (Meier et al., 2012; Rüffel et al., 2018). However, whether these biominerals have unique physiochemical properties compared with abiogenic minerals remain to be investigated. Previous studies on aSRB tend to separate the site of microbial activity (e.g., H_2_S generation) and metal precipitation due to the focus on metal recovery. Studies on nSRB in which microbial activity and mineral precipitation are closely associated have shown that biogenic MeS tend to display higher crystallinity than their abiogenic counterparts (Mansor, Berti, et al., 2019; Mansor, Winkler, et al., 2019; Mansor & Xu, 2020; Parigi, Chen, et al., 2022; Parigi, Pakostova, et al., 2022; Picard et al., 2018; Xu et al., 2016). Furthermore, cell‐derived organic carbon can associate strongly and be preserved with biogenic MeS (Nabeh et al., 2022; Picard et al., 2019; Truong et al., 2023). It has been proposed that ZnS can replace organic tissue and algae filaments in acidic pit lakes, preserving their morphology (Ilin et al., 2022). Thus, more comprehensive studies on biogenic minerals produced by aSRB and their associated carbon may be promising avenues for identifying biosignatures.

Here, it is worth considering if the formation of specific minerals that seem to be precipitated in disequilibrium with the surrounding environments could be used as a biosignature. For example, the formation of siderite (FeCO_3_) in the acidic Río Tinto system was completely unexpected from the viewpoint of bulk geochemistry. Experimental studies attributed the formation of siderite due to the generation of high pH and high Fe^2+^ around aFeRB (Sánchez‐Román et al., 2014). Acidophilic SRB can also create microenvironments with different pH, CO_2_, activity, and H_2_S concentrations than the bulk solution (see reactions in Table 2), potentially leading to the formation of unexpected minerals under certain geochemical conditions (such as siderite) that could be useful as biosignatures.

Neutrophilic SRB are known to produce large sulphur isotopic fractionation (δ^34^S) during sulphate reduction, with sulphide being depleted relative to sulphate by up to 66 ‰ (Sim et al., 2023). This large fractionation, when detectable in co‐existing sulphide and sulphate minerals in the geological record, is considered as strong evidence for biosignatures (Moreras‐Marti et al., 2022). The isotopic fractionation induced by aSRB during sulphate reduction has not been studied and it is unclear if pH will have an effect. The enzymes involved in sulphate reduction are highly conserved (Sim et al., 2023) and so far, there is no known difference with the mechanisms of sulphate reduction at low versus neutral pH. Furthermore, the cell‐specific sulphate reduction rate—an important parameter that correlates with apparent isotopic fractionation—seems to be similar for both aSRB and nSRB (Sánchez‐Andrea et al., 2014). Hence there is a priori no reason to suspect that their sulphur isotopic fractionation will be different.

In addition to S isotopes, the fractionation of non‐traditional stable isotopes should also be considered. Recently, it was shown that Ni sulphides precipitated in the presence of nSRB are about 1‰ lighter in Ni isotopes (δ^60^Ni) than abiogenic NiS (Parigi, Chen, et al., 2022; Parigi, Pakostova, et al., 2022). This is in contrast to Fe isotopes (δ^56^Fe), in which the isotopic fractionation for biological versus abiotic processes tend to overlap (Johnson et al., 2020). Thus, different isotopic systems have different biosignature potentials.

In summary, little is known about the potential biosignatures produced by aSRB at the moment. Previous studies on nSRB have shown promising biosignatures in the form of biominerals and stable isotopes. Similar studies on aSRB will enrich our understanding not only for biosignatures but also for understanding the impact of microbial processes occurring daily in acidic environments on Earth.

## CONCLUDING REMARKS

Acidophilic SRB are present and even dominant in low‐pH and high metal‐content environments (e.g., acidic pit lakes with pH<5). They employ several mechanisms to maintain cell homeostasis (e.g., proton exclusion and Donnan potential). Thermodynamic calculations showed that aSRB have access to energetically favourable metabolisms to compensate the high energy demand of living under these extreme conditions. Nevertheless, issues related to ATP generation during electron‐transport phosphorylation (proton intrusion) and cell inhibition by waste products at low pH (e.g., acetate and hydrogen sulphide) remain as challenges to these microorganisms. Interactions with other microbial groups strongly define aSRB's roles in acidic environments. Positive interactions (e.g., syntrophy) likely enhance the survival of aSRB together with other microbial groups, thus expanding their metabolic networks in the whole community. Under non‐favourable conditions (e.g., extremely low pH), aSRB likely rely on pioneer microbial populations to survive, forming a commensalism‐like interaction with fermentative microorganisms. In addition, aSRB could be outcompeted by other microorganisms (e.g., FeRB) for specific electron donors, thus affecting their establishment into the environment. Biological treatment of AMD using aSRB is one of the most sustainable options available and could be enhanced when a microbial consortium is used instead of monocultures. Selective recovery of metals via biosulphidogenesis and its recycling for technological applications (e.g., metal sulphides as quantum dots) is also an emergent process that could contribute to the establishment of circular economy. Biosignatures based on aSRB remain an underexplored area of research with a profound impact to our search for life in the universe. Acidophilc SRB is a key microbial group in acidic and metalliferous environments and their adaptations and metabolic features give them a pivotal place in biogeochemical cycles and in technological applications.

## AUTHOR CONTRIBUTIONS

**Luis Felipe Valdez‐Nuñez:** Conceptualization; project administration; writing – original draft; writing – review and editing; visualization. **Andreas Kappler:** Conceptualization; formal analysis; validation; writing – review and editing; funding acquisition. **Diana Ayala‐Muñoz:** Conceptualization; investigation; writing – original draft; writing – review and editing. **Idelso Jamín Chávez:** Investigation; methodology; software; visualization. **Muammar Mansor:** Conceptualization; supervision; data curation; validation; writing – original draft; writing – review and editing.

## CONFLICT OF INTEREST STATEMENT

The authors declare no conflicts of interest.

## Data Availability

Data sharing is not applicable to this article as no new data were created or analysed in this study.
